# Evaluation of Interleukin-10, Vascular Endothelial Growth Factor Levels, and Bone Marrow Parameters in Multiple Myeloma Patients at Diagnosis and After Treatment

**DOI:** 10.3390/diagnostics15131641

**Published:** 2025-06-27

**Authors:** Fulya Memis, Meryem Yalvac Kandefer, Sonay Aydin, Klara Dalva, Selami Kocak Toprak

**Affiliations:** 1 Department of Internal Medicine, Faculty of Medicine, Erzincan Binali Yıldırım University, Erzincan 24100, Turkey; meryemyalvac@hotmail.com; 2 Department of Radiology, Faculty of Medicine, Erzincan Binali Yıldırım University, Erzincan 24100, Turkey; sonay.aydin@erzincan.edu.tr; 3 Department of Hematology, Faculty of Medicine, Ankara University, Ankara 06620, Turkey; dalva@ankara.edu.tr (K.D.); sktoprak@yahoo.com (S.K.T.)

**Keywords:** monoclonal gammopathy, angiogenesis, mobilization, plasma cells, international staging system

## Abstract

**Background:** Interleukin-10 (IL-10) and vascular endothelial growth factor (VEGF) are believed to possess a role in the pathophysiology of multiple myeloma (MM). We aimed to assess the significance of these parameters in the diagnosis, monitoring, and prognosis of the disease by examining them in patients at diagnosis and post-treatment and comparing the findings with those of healthy individuals. **Methods:** We conducted blood sampling from 35 patients diagnosed with MM at the time of diagnosis and from 15 of these patients post-treatment. We additionally assessed similar serum markers in a control group of 15 healthy individuals. Furthermore, we documented laboratory results, organ involvement, comorbidities, and CD27-CD81 levels assessed using flow cytometry in the bone marrow, along with treatments and patient responses. We also examined the quantity of cells collected during mobilization in patients who had autologous stem cell transplantation. **Results:** We found a positive correlation (*p* = 0.028/*p* = 0.035) between IL-10 and VEGF with the international staging score. In patients with renal involvement, IL-10 levels were higher and VEGF levels were lower than those without renal involvement (*p* = 0.011/*p* = 0.012). We showed that VEGF levels decreased significantly with treatment (*p* = 0.001). We found no statistically significant correlation between treatment responses and IL-10 and VEGF. The number of CD34 cells collected by mobilization showed a negative correlation with CD27 and a positive correlation with VEGF (*p* = 0.007/*p* = 0.032). **Conclusions:** Serum IL-10 level is associated with ISS and renal involvement in MM patients. There is a positive correlation between serum VEGF levels and the number of stem cells collected during mobilization. As CD27 expression increases, the number of stem cells collected in mobilization decreases.

## 1. Introduction

Multiple myeloma (MM) is a malignant disease characterized by the proliferation of clonal plasma cells in the bone marrow, typically releasing monoclonal immunoglobulins detectable in serum or urine. This disease, typically manifesting in old age, is characterized by monoclonal immunoglobulin synthesis, plasma cell accumulation in the bone marrow, and organ damage, as shown by the CRAB criteria: hypercalcemia, renal failure, anemia, and bone lesions [1]. Substantial data indicates that the bone marrow microenvironment significantly influences the neoplastic growth of plasma cells [2]. Current studies assessing the relationship between MM cells and the bone marrow microenvironment emphasize cell–cell and/or cell–matrix interactions, as well as growth factors and cytokines [1,3].

In this context, bone marrow stromal cells, immune cells, and cytokines released by these cells play crucial roles in the beginning and progression of MM. The interaction between tumor cells and the microenvironment influences disease prognosis via extracellular signals and soluble substances. Particularly interleukins (including IL-6), tumor necrosis factor-alpha (TNF-α), and growth factors facilitate the proliferation of MM cells and the emergence of drug resistance [3,4,5].

Interleukin-10 (IL-10) is a cytokine produced by monocytes/macrophages, T and B lymphocytes, natural killer (NK) cells, and mast cells [4]. IL-10 stimulates the proliferation of B cells and causes an increase in plasma cells [5]. In this context, it functions as a growth factor for MM cells [6]. MM cells become more sensitive to IL-10 due to the autocrine release of oncostatin M production. This interaction is thought to be the mechanism of IL-10 activity on MM cells [5,6].

Researchers have identified angiogenetic factors that induce tumor cell proliferation and disease progression [6]. Vascular endothelial growth factor (VEGF) is the primary angiogenic factor responsible for angiogenesis [7]. VEGF, released in response to IL-6, serves as a potent growth factor for malignant cells and is crucial for vascularization. The paracrine relationship that exists between VEGF and IL-6 is thought to be important to a complex regulatory system that maintains tumor growth and protects MM cells against apoptosis [6,7]. VEGF levels in the bone marrow of MM patients were markedly elevated compared to those in monoclonal gammopathy of undetermined significance (MGUS) and healthy individuals [8].

Flow cytometry shows that neoplastic plasma cells have positive results for CD138 (Cluster of Differentiation 138) and CD38 antibodies, unusual positive results for CD56, and negative results for CD19 [9]. Recent studies have highlighted that notable alterations in the expression of many surface antigens, including CD27 and CD81, have been detected in neoplastic plasma cells. The prevailing consensus is that the loss of CD27 and greater expression of CD81 are correlated with poor prognosis [10,11].

This study aimed to assess the baseline values of the aforementioned parameters at diagnosis and post-treatment in MM patients, compare them with those of a healthy population, and evaluate their significance in diagnosis, follow-up, and prognosis of the disease. In contrast to the majority of studies published in the literature, the goal of our study was to investigate the alterations in the parameters assessed with MM treatment and their efficacy in predicting treatment response.

## 2. Materials and Methods

### 2.1. Ethical Aspects

This study received ethical clearance from the Clinical Studies Ethical Committee at Ankara University School of Medicine (Protocol number: 03-122-17/Date: 13 February 2017). All patients provided signed informed consent for publication of this study utilizing their data.

### 2.2. Study Population

From October 2017 to October 2018, 35 patients diagnosed with multiple myeloma at our hospital were included in our study, and peripheral blood samples were collected prospectively at diagnosis and post-treatment periods.

Our study included adult patients diagnosed with MM within the specified time frame whose treatment was completed and follow-up carried out at our center. Patients diagnosed with MM who received treatment at another location and those who died were excluded from the study due to the unavailability of post-treatment outcomes.

During the specified time frame, post-treatment samples were unattainable due to 15 patients discontinuing follow-up at our center and 5 patients dying due to their conditions. These twenty patients could only be sampled at the time of diagnosis, and these patients were evaluated with the data at the time of diagnosis. We assessed fifteen individuals using data collected both at the time of diagnosis and after treatment. The healthy control group comprised fifteen entirely healthy people, matched in age and gender to the MM patient group. Identical peripheral blood samples were collected from the individuals in this group.

In our patient cohort, post-treatment sampling occurred 6 months post-transplantation for those who underwent autologous stem cell transplantation, 6 months after maintenance therapy for patients receiving maintenance treatment, and 6 months following treatment for those who completed a treatment regimen.

### 2.3. Peripheral Blood Sampling

Venous blood samples were obtained from patients (35 at diagnosis and 15 after treatment) and a healthy control group. Venous blood samples were collected a single time from healthy individuals. The blood samples underwent centrifugation, and serum samples were preserved at −20 °C. All samples were analyzed at the end of the study to reduce variability. Measurements of IL-10 and VEGF were conducted using the ELISA method (Quantikine, R&D Systems Inc., Minneapolis, MN, USA) in accordance with the manufacturer’s guidelines. The amounts of IL-10 and VEGF in the serum were measured using a standard curve created with known IL-10 and VEGF proteins.

### 2.4. Disease Severity and Comorbidities

Our patients were categorized into three stages based on the International Staging System (ISS), the most prevalent method among various risk stratification systems established for MM [12].

We documented the demographic data, including age and sex, at the time of diagnosis for all patients in our study. We also assessed and classified the serum lactate dehydrogenase (LDH) and beta 2-microglobulin (B2M) levels as normal or increased, according to the laboratory’s upper limit. The biochemistry laboratory at our center established the highest-level limits as 247 U/L for LDH and 3.47 mg/dL for B2M.

The ISS stages of the patients were checked, and they looked for any issues with the bones, nervous system, and kidneys (creatinine ≥ 2). The study incorporated the assessment of CD27 and CD81 levels from bone marrow samples via flow cytometry.

Along with the aforementioned factors, the existence of diabetes mellitus (DM), hypertension (HT), smoking, risk of cytogenetic abnormalities, first-line treatments and how they respond, as well as additional treatments and responses six months following the evaluation of these treatments in 15 patients whose follow-up was completed. We also assessed the number of cells mobilized in patients who underwent transplantation.

### 2.5. Statistical Analysis

The data were analyzed using the Package for Social Sciences (SPSS) 25 for Windows (IBM SPSS Inc., Chicago, IL, USA). The normal distribution of the data was evaluated using the Kolmogorov–Smirnov test. All the variables revealed non-normal distribution. We display the variables that do not follow a normal distribution as median (minimum–maximum) values. We display categorical variables as numbers and percentages. For the comparison of median IL-10 and median VEGF values between patient feature subgroups and patient/healty control groups, the Mann–Whitney U test was used. For the comparison of IL-10 and VEGF values according to treatment, the Wilcoxon test was used. The Kruskal–Wallis test was applied for the comparison of IL-10, VEGF, CD27, and CD81 values amongst treatment response subgroups. A two-tailed value of *p* <  0.05 was considered statistically significant. Spearman analysis was used to check how IL-10 and VEGF values relate to ISS grade and mobilization. To define the cut-off values of IL-10 and VEGF for predicting disease presence, ROC analysis was performed.

## 3. Results

The study comprised 35 MM patients and 15 healthy individuals as the control group. Fifteen patients had final samples acquired post-treatment, while twenty patients did not have their final samples obtained following treatment. Among the 35 patients, 54.2% were female (19 females, 16 males), with a median age of 65.75 (range 40–89) years (Table 1).

All patients in the study were divided into two groups based on their gender, whether they had high or normal LDH and B2M levels, and if they had issues with their bones, nervous system, or kidneys. Their levels of IL-10 and VEGF were checked (Table 2). Gender, serum LDH levels, and the presence of bone and nervous system involvement had no correlation with IL-10 and VEGF levels. Moreover, patients presenting elevated serum B2M had greater levels of IL-10 and lower levels of VEGF; nevertheless, these differences did not achieve statistical significance (*p* = 0.113/*p* = 0.102).

The median IL-10 level was 14.17 pg/mL in patients with renal involvement and 7.54 pg/mL in those without renal involvement. In patients with renal involvement, the median VEGF level was 124.46 pg/mL, while in patients without renal involvement, it was 295.28 pg/mL. The presence of renal involvement was statistically significantly associated with elevated IL-10 and reduced VEGF levels in serum (*p* = 0.011/*p* = 0.012).

**Figure 1:**
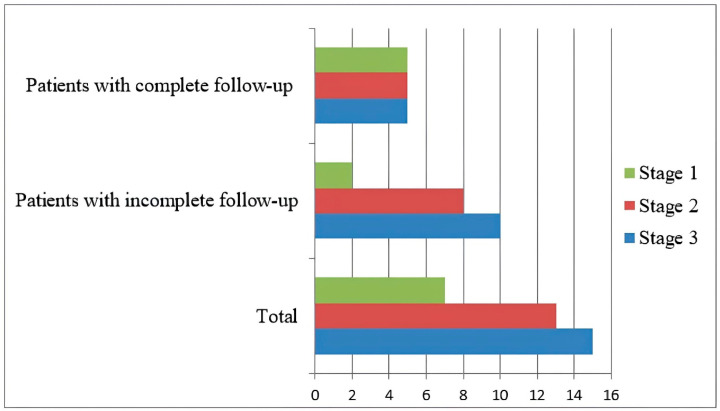
Patient stages according to the International Staging System.

According to the International Staging System (ISS), seven patients were classified as stage 1, thirteen as stage 2, and fifteen as stage 3 at the time of diagnosis (Figure 1).

IL-10 and VEGF levels at diagnosis were analyzed in relation to ISS stages among all patients. IL-10 levels were reported to increase, while VEGF levels decreased with advancing ISS stage (Table 3). A positive connection existed between ISS and IL-10, while a negative correlation was observed between ISS and VEGF.

In our study group of 15 patients with completed follow-up, categorization was performed based on the features presented in Table 2, and the serum levels of IL-10 and VEGF at the time of diagnosis were calculated. However, significant statistical findings were unattainable due to the imbalance in group distributions and the inadequacy of the population. There was no meaningful difference in the levels of IL-10 and VEGF when we looked at patients in this group based on whether they had diabetes, high blood pressure, smoked, or had certain genetic risks.

No meaningful difference was found in all patients (*n* = 35) when looking at the relationship between serum IL-10 and VEGF levels at diagnosis and the baseline levels of CD27 and CD81.

Table 4 displays the treatment options and responses of patients who have finalized their follow-up. Eleven out of the fifteen patients underwent autologous stem cell transplantation (ASCT). The advanced age and inadequate performance condition in three patients prevented the scheduling of ASCT. One patient received allogeneic stem cell transplantation (allo-SCT) following seven cycles of VTD (bortezomib + thalidomide + dexamethasone) due to a diagnosis of primary immunodeficiency rather than ASCT. Table 4 demonstrates the types of mobilization preparation and the quantity of CD34 cells harvested in patients who also received ASCT. In eight cases, only Granulocyte Colony-Stimulating Factor (G-CSF) was utilized for cell mobilization. In the remaining three patients, supplementary medications were administered alongside G-CSF.

In 11 patients who had ASCT, we looked at how the number of CD34 cells collected during mobilization related to the levels of IL-10, VEGF, CD27, and CD81 at the time of diagnosis, and the results are shown in Table 5. The quantity of the collected cells diminished with an increase in the CD27 ratio, while the quantity of the collected cells increased with rising VEGF levels (*p* = 0.007/*p* = 0.032). The quantity of the cells collected showed no statistically significant difference between IL-10 and CD81 (Spearman’s rho).

Upon comparing the IL-10 and VEGF levels of patients at diagnosis with those from the healthy control group, it was notable that the median IL-10 level was elevated in the patient cohort (Table 6), albeit without reaching statistical significance (*p* = 0.061).

The IL-10 and VEGF values at diagnosis among patients with completed follow-up were compared to those post-treatment (Table 7). The levels of IL-10 and VEGF in the patients reduced after treatment. A statistically significant reduction in VEGF levels at diagnosis was seen post-treatment (*p* = 0.001).

There was no statistically significant distinction seen between IL-10 and VEGF levels for first-line and final treatment responses, as determined by Kruskal–Wallis analysis. Comparing CD27 and CD81 values based on treatment responses revealed that individuals with elevated CD27 levels exhibited a greater complete response (CR) compared to those with a very good partial response (VGPR) as the initial treatment response (*p* = 0.004). No association existed between final stage responses and the values of CD27 and CD81.

**Figure 2:**
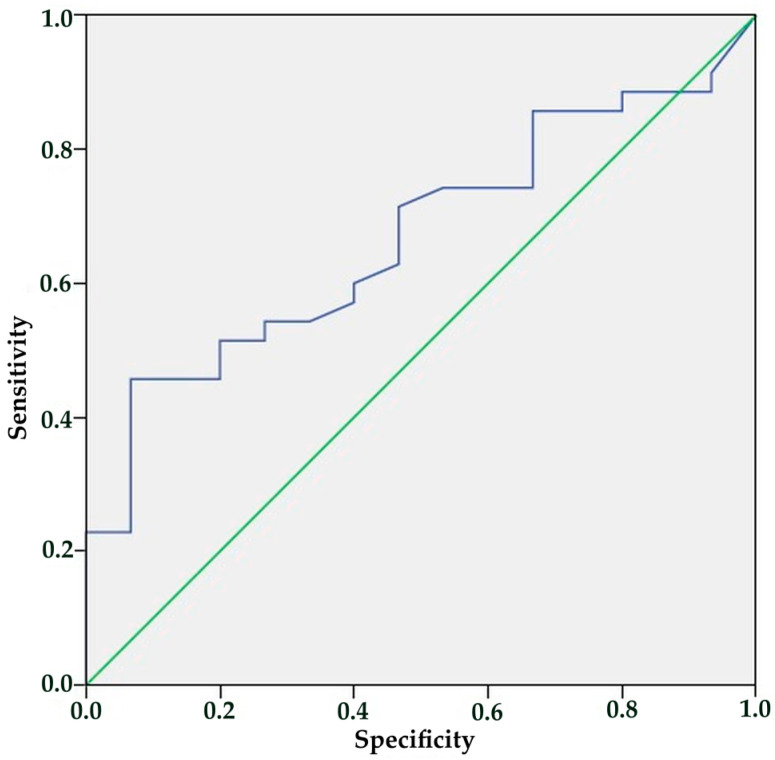
ROC curve for IL-10.

The ROC curve analysis showed that IL-10 and VEGF levels were not useful for diagnosis because there was no clear cut-off for VEGF, as patients and healthcare experts had similar average levels. The diagnostic cut-off value for IL-10 was determined to be 4.41 pg/dL, while exhibiting low sensitivity and specificity rates. The sensitivity and specificity were 71.4% and 53.3%, respectively. Figure 2 presents the ROC curve.

## 4. Discussion

Studies on IL-10, a potent B cell differentiation factor and presumed growth factor for neoplastic plasma cells, with VEGF, the primary angiogenesis growth factor associated with the progression of MM, indicate that these parameters correlate with disease stage and prognosis [13,14,15,16]. This study was carried out to evaluate MM patients during diagnosis and post-treatment, comparing them with healthy individuals, considering the significance of these parameters in diagnosis, follow-up, and prognosis.

### 4.1. Relationship Between IL-10, VEGF and ISS

The role of IL-10 within the tumor milieu remains uncertain; nonetheless, studies indicate that both VEGF and IL-10 levels are elevated in multiple myeloma relative to healthy individuals and rise concurrently with the ISS stage [12,17].

Alexandrakis et al. [13] compared the levels of serum IL-10 and VEGF in 54 multiple myeloma patients and healthy people, finding that IL-10 was twice as high and VEGF levels were also much higher in the patients. In our research, IL-10 levels were elevated in patients relative to healthy individuals; however, this difference lacked statistical significance. A study by Wang et al. [14] demonstrated that serum IL-10 levels were significantly elevated (ten times) in MM patients compared to healthy individuals. Both analyses indicate that the difference in serum IL-10 levels between patients and healthy individuals becomes more severe with a greater number of participants in the study. The recurrent finding of increased IL-10 levels in MM patients across various investigations, including our own, highlights its potential as a diagnostic or prognostic marker. Although our cohort of 35 patients did not demonstrate statistical significance, the observed twofold increase in IL-10 levels corresponds with the findings of Alexandrakis et al. [13] and Wang et al. [14], indicating that IL-10 upregulation is likely a prevalent characteristic in MM. IL-10 is an immunosuppressive cytokine, meaning it helps the immune system tolerate the tumor environment, which could influence how well treatments work. Future studies should aim to elucidate the thresholds for clinically significant IL-10 increase and examine its potential as a treatment target or stratification marker.

Alexandrakis et al. [13] also demonstrated elevated IL-10 and VEGF levels in advanced ISS stages. Wang et al. [14] have shown that IL-10 levels are elevated in advanced ISS stages. The increase in IL-10 and VEGF levels in advanced ISS stages shows that as the disease becomes worse, there is more immune suppression and blood vessel growth happening in the tumor environment. In our study, the significant correlation between IL-10 levels and ISS stage (*p* = 0.028) highlights the importance of this immunomodulatory cytokine in the pathophysiology of MM and suggests that it may serve as a marker of disease burden or activity. The increased immunosuppressive effect of IL-10 might explain why patients in later stages have a worse outlook and could be seen as a possible target for specific immunotherapies.

In contrast to the study carried out by Alexandrakis et al., our findings indicated that VEGF levels decreased in advanced ISS stages (*p* = 0.035). Sezer et al. [16] found no association between serum VEGF levels and MM stage in a study involving 56 MM patients, 11 individuals with MGUS, and 20 healthy controls. It was proposed that VEGF could be released from active platelets throughout the assessment; thus, platelet-poor plasma should be utilized for VEGF quantification [18,19].

Although prior research has indicated that VEGF levels are markedly increased in MM patients, typically correlating with the disease stage, our findings were only partially aligned with this pattern. We suggest that these outcomes stem from a possible adverse impact of our limited sample size. Our discovery of a negative correlation between VEGF levels and ISS stages contradicts other results and prompts significant inquiries regarding the dependability of blood VEGF as a biomarker for disease progression. This discrepancy may arise from pre-analytical variables, including the sample type utilized.

### 4.2. Relationship Between IL-10, VEGF and Patient Characteristics

In our study, we split all patients into two groups based on gender, LDH and B2M levels, and whether they had issues with their bones, nervous system, or kidneys, and then we compared the levels of IL-10 and VEGF.

We observed that IL-10 levels were elevated and VEGF levels decreased in patients who had higher B2M levels; however, these findings failed to reach statistical significance. Shekarriz et al. [20] demonstrated a significant positive correlation between elevated IL-10 levels and B2M concentrations. Furthermore, they proposed the assessment of serum IL-10 levels as a noninvasive diagnostic test to ascertain disease severity in MM patients, although they acknowledged that the evaluation of serum IL-10 levels post-treatment was a limitation of their research. Our study demonstrated a reduction in serum IL-10 levels in post-treatment measurements, albeit not significantly. The research carried out by Valkovic et al. [21] identified a relationship between VEGF and disease stage, but no significant association was found with B2M. Alexandrakis et al. [13] observed an advantageous correlation between VEGF and B2M. Sezer et al. [16] found a non-significant negative connection between VEGF and B2M. Our study results revealed that VEGF levels decreased in patients with higher B2M; however, this difference lacked statistical significance. The conflicting results of these studies cast doubt on the reliability of VEGF as a prognostic marker in MM patients.

These findings once more expose the discrepancies in the current literature concerning the correlation between B2M, IL-10, and VEGF. While IL-10 levels are considered promising for diagnostic and prognostic purposes, the limits in assessing post-treatment changes raise concerns about the reliability of this biomarker. The association between VEGF and B2M exhibits variability in direction and significance across several studies. Therefore, more future studies with larger groups of patients that include the treatment process are needed to assess how useful IL-10 and VEGF are for predicting outcomes in MM.

In our study, IL-10 levels were markedly elevated in patients with renal involvement relative to those without renal involvement. Conversely, VEGF levels were significantly reduced in patients with renal involvement. The differences were statistically significant (*p* = 0.011/*p* = 0.012). Contrary to our findings, Wróbel et al. [22] observed elevated VEGF levels in patients with renal insufficiency (creatinine levels ≥ 2 mg/dL) relative to those with normal renal function in their study. Upon reviewing the literature, we discovered no additional research concerning IL-10 and its involvement in renal issues related to MM. Moreover, studies indicating that elevated serum IL-10 levels correlate with renal failure have captured our interest [23,24]. Our research underscores the possible involvement of these cytokines in the development of renal failure in MM. Elevated IL-10 may exacerbate disease by inhibiting pro-inflammatory responses or directly inducing damage to renal tissue. This study suggests that IL-10 ought to be regarded as a unique biomarker for predicting or monitoring renal failure in MM patients, in addition to being a possible candidate for targeted therapy as previously indicated.

The decrease in VEGF levels in individuals with renal involvement contradicts the findings of Wróbel et al. [22], which indicate that VEGF is enhanced in renal failure. This differing observation shows that VEGF’s role in MM might change depending on different stages of kidney involvement or different disease processes. Reduced VEGF levels in the context of renal injury may signify a lack of angiogenesis or a disruption in vascular healing mechanisms, thereby exacerbating the course of renal injury. Since there is not much research on how IL-10 directly relates to kidney issues in MM, our study’s results, which clearly show this connection, are important for filling the knowledge gap in this area. Future research should look more closely at how these cytokines work in the kidneys, how they differ in various groups of patients, and how they could help with tracking the disease and treatment choices in medical practice.

### 4.3. Relationship Between IL-10/VEGF and Treatment Response

A study conducted by Wang et al. [14] involving 188 MM patients revealed that 66 individuals received chemotherapy along with ASCT, while 122 patients underwent chemotherapy exclusively. The findings indicated that patients who had lower IL-10 levels exhibited markedly greater CR compared to those with higher IL-10 levels. A study by Pour et al. [25] revealed that individuals exhibiting CR and VGPR had considerably lower levels of VEGF. We demonstrated that baseline serum IL-10 and VEGF levels at diagnosis were elevated in individuals exhibiting a first-line therapy response of PR, while post-treatment IL-10 levels were lowest in patients achieving CR in our study. Likewise, we observed that post-treatment serum IL-10 levels were the lowest in patients exhibiting a CR to treatment. However, owing to our limited patient population, the correlation between treatment responses and serum IL-10 and VEGF levels did not attain statistical significance. Nonetheless, based on the existing literature, the current data indicate that IL-10 may offer clues about treatment response. The possible relationship between IL-10 levels and treatment success, particularly the reduced IL-10 levels noted in patients exhibiting CR, has garnered significant interest in the literature. When assessed in conjunction with existing literature, IL-10 is considered a potential indicator of both disease activity and treatment response. Higher levels of VEGF are linked to lower response levels, suggesting that these markers could be important for predicting outcomes. The restricted patient sample in our study precluded the attainment of statistical significance. Consequently, additional research involving bigger sample sizes, multicenter approaches, and comparisons of pre- and post-treatment levels is necessary to elucidate the efficacy of IL-10 and VEGF levels in forecasting treatment response.

Tarin et al. [26] correlated the loss of CD27 and an increase in CD81 expression with relapse in a study involving MGUS and MM patients. Furthermore, the expression of CD27 and CD81 in patients exhibiting a CR was similar to that observed in patients with MGUS. Upon comparing CD27 and CD81, which are believed to be associated with disease progression, to treatment responses, we demonstrated that a CR was more frequently observed in patients exhibiting higher CD27 levels (*p* = 0.004). Given these results, we propose that CD27, a significant factor influencing treatment response in MM patients, belongs to the TNF receptor family, which is essential for B cell growth and activation. We hypothesize that elevated CD27 levels may contribute to the development of tumor cells with a less aggressive phenotype or enhance immune system-mediated tumor control. Future prospective cohorts are required to assess milder or more aggressive treatments according to patients’ baseline CD27 levels.

The initial treatment result was PR in two patients with DM. Upon reviewing the literature on this subject, we identified studies indicating that DM had no impact on MM survival; however, we could not find any studies assessing its effect on treatment responsiveness [27,28]. We propose that DM may be a comorbidity requiring investigation in patient follow-up due to its potential impact on treatment response.

Numerous prior research studies have demonstrated that serum B2M levels may predict the results of treatment [29,30]. The response to first-line treatment was predominantly VGPR in patients with elevated serum B2M, while CR was primarily observed in those with normal serum B2M (*p* = 0.03). These results confirm the correlation between B2M and disease burden, as well as a poor prognosis. The higher rates of CR in patients with normal B2M levels suggest that B2M could help identify patients who are diagnosed early or have better chances of recovery. Thus, evaluating B2M levels in treatment planning may be important in forecasting patient prognosis.

The final serum IL-10 and VEGF levels of 15 patients who completed the follow-up post-treatment were elevated compared to the healthy control group; however, this difference lacked statistical significance. Furthermore, serum levels of IL-10 and VEGF decreased post-treatment in comparison to the baseline values of the patients. The reduction observed for VEGF was significant (*p* = 0.001). Iwasaki et al. [31] observed a notable reduction in VEGF levels with effective chemotherapy while indicating that VEGF levels remained unchanged in the absence of therapeutic response. These findings underscore the significance of VEGF for assessing treatment response. VEGF may serve as a biomarker for monitoring treatment response. Additionally, the observed drop in IL-10 levels post-treatment in our study, although not statistically significant, suggests that IL-10 could potentially indicate a response to treatment.

### 4.4. Relationship Between IL-10, VEGF and Mobilization

The correlation between the quantity of CD34 cells harvested during mobilization and the levels of IL-10 and VEGF, along with the expression of CD27 and CD81, was investigated in 11 patients who underwent ASCT. A negative association was observed between CD34 stem cell count and CD27 expression (*p* = 0.007), while a positive correlation was noted with VEGF levels (*p* = 0.032). The findings of our research indicate, as noted by Chhabra et al. [32], that elevated basal VEGF levels forecast effective stem cell mobilization.

In our study, we observed that the number of cells collected in stem cell mobilization decreased as CD27 expression increased. No studies investigating the effect of CD27 on mobilization were found in the literature. Our findings, which for the first time in the existing literature elucidate the possible impact of CD27 expression on stem cell mobilization, are notably significant in this regard. Elevated levels of CD27 markedly decreased the quantity of mobilized CD34+ cells, indicating that this molecule could have a suppressive influence on hematopoietic stem cell dynamics. On the other hand, our study found that higher levels of VEGF are linked to better success in mobilization, supporting the results of Chhabra et al. [32] and suggesting that VEGF might play a role not just in blood vessel formation but also in managing the environment for blood cell development. These original discoveries significantly contribute to the literature, particularly as the initial demonstration of CD27’s effect on mobilization, and they will illuminate further studies investigating advanced molecular and cellular mechanisms.

### 4.5. Strengths and Limitations of the Study

This detailed study looked at lab results, how different organs were affected, other health issues, CD27–CD81 levels measured with flow cytometry in bone marrow, treatment plans, how well treatments worked, and the number of stem cells collected from patients who had ASCT. Pre- and post-treatment data had been compared with a healthy control group, and the interactions of all these variables with IL-10, VEGF, CD27, and CD81 were evaluated within an isolated research group.

The most significant limitation of our study is its single-center design and the limited patient cohort. The limited sample size (35 patients) may undermine the generalizability and reliability of certain significant results and implications. We are preparing a multicenter investigation including a larger population. We believe our article will encourage extensive cohort studies on these topics. We believe that extensive cohort studies published by various centers will corroborate our findings.

## 5. Conclusions

In MM patients, serum IL-10 levels increase with advancing ISS stages. There is no substantial decrease with treatment, nor is it correlated with treatment responses. Moreover, renal involvement is more prevalent in patients with elevated IL-10 levels.

The serum VEGF levels decrease with treatment in MM patients; however, that decrease is not correlated with treatment response. A beneficial correlation exists between serum VEGF levels and the quantity of stem cells harvested via mobilization.

Flow cytometry assessments of CD27 concentrations lead to a CR rather than a VGPR. CD27 is correlated with the quantity of stem cells harvested during mobilization. As CD27 expression grows, the quantity of stem cells harvested during mobilization falls.

In conclusion, the association between IL-10 and renal involvement, as well as CD27 and stem cell quantity, is significant, necessitating further comprehensive studies to clarify these interactions.

## Figures and Tables

**Table 1:** Demographic distribution, clinical and laboratory findings of patients and healthy group.

	Patients with Incomplete Follow-Up (*n* = 20)	Patients with Complete Follow-Up (*n* = 15)	Healthy Control Group (*n* = 15)
Sex *n* (%)			
Female	11 (55)	8 (53.3)	8 (53.3)
Male	9 (45)	7 (46.7)	7 (46.7)
Median age (min–max)	69.3 (40–89)	62.3 (44–85)	62.3 (44–85)
Laboratory findings			
High LDH levels *n* (%)	6 (30)	1 (6.6)	
High B2M levels *n* (%)	12 (60)	9 (60)	
Involving bone *n* (%)	6 (40)	13 (86.6)	
Involving nervous system *n* (%)	3 (15)	3 (20)	
Involving kidney *n* (%)	7 (35)	2 (13.3)	
Cigarette smoker *n* (%)		5 (33.3)	
Presence of DM *n* (%)		2 (13.3)	
Presence of HT *n* (%)		6 (40)	

LDH: lactate dehydrogenase; B2M: Beta 2-microglobulin; DM: diabetes mellitus; HT: hypertension.

**Table 2:** Patient IL-10 and VEGF levels by gender, laboratory findings, and organ involvement at diagnosis.

Patient Features (*n* = 35)	Median IL-10 (pg/mL)	*p* Value *	Median VEGF (pg/mL)	*p* Value *
Female (*n* = 19)	9.26	0.987	247.06	0.518
Male (*n* = 16)	9.21		256.46	
Elevated LDH levels (*n* = 7)	9.77	0.772	250.02	0.635
Normal LDH levels (*n* = 28)	9.10		251.68	
Elevated B2M levels (*n* = 21)	10.63	0.113	205.35	0.102
Normal B2M levels (*n* = 14)	7.15		320.35	
With bone involvement (*n* = 19)	9.50	0.934	239.54	0.882
Without bone involvement (*n* = 16)	8.92		265.38	
With nervous involvement (*n* = 6)	8.93	0.965	280.06	0.793
Without nervous involvement (*n* = 29)	9.30		245.41	
With kidney involvement (*n* = 9)	14.17	0.011	124.46	0.012
Without kidney involvement (*n* = 26)	7.54		295.28	

IL-10: Interleukin-10; VEGF: vascular endothelial growth factor; LDH: lactate dehydrogenase, B2M: Beta 2-microglobulin. * *p* value was obtained from Mann–Whitney U test.

**Table 3:** IL-10 and VEGF relationship at diagnosis with International Staging System (Spearman’s rho).

ISS	IL-10	VEGF
Correlation coefficient	0.371	−0.357
Sig. (2-tailed)	0.028	0.035

IL-10: Interleukin-10; VEGF: vascular endothelial growth factor; ISS: International Staging System.

**Table 4:** Treatments given to patients whose follow-up was completed and their responses, cell mobilization, and CD34 cell counts in patients who underwent autologous blood stem cell transplantation; ^1^ VCD: Bortezomib/Cyclophosphamide/Dexamethasone; ^2^ VTD: Bortezomib/Thalidomide/Dexamethasone; ^3^ VMP: Bortezomib/Melphalan/Prednisone; ^4^ VGPR: very good partial response/PR: partial response/CR: complete response; ^5^ ASCT: autologous blood stem cell (PBSC) transplantation; ^6^ current situation: responses during the sampling process six months post-transplantation in patients undergoing transplantation, six months post-transplantation in patients on maintenance therapy, and six months post-treatment in patients undergoing a treatment regimen; ^7^ G-CSF: granulocyte-colony stimulating factor; ^8^ unit of the number of cells collected: ×10^6^/kg.

Patient Number	First Line Treatment	First-Line Treatment Response	Additional Treatment	Additional Treatment Response	Current Situation ^6^	Pre-ASCT Cell-Collection Drug	Pre-ASCT Cell Count ^8^
1	4 cycles VCD ^1^	VGPR ^4^	ASCT ^5^	CR	CR	G-CSF ^7^	9.21
2	4 cycles VCD	PR ^4^	ASCT	VGPR	PR	G-CSF	6.07
3	4 cycles VCD	VGPR	ASCT	VGPR	VGPR	G-CSF + Cyclophosphamide + Plerixafor	8.81
4	4 cycles VCD	CR ^4^	ASCT	CR	CR	G-CSF	6.45
5	3 cycles VCD	VGPR	ASCT	CR	CR	G-CSF + Cyclophosphamide	11.3
6	4 cycles VCD	VGPR	ASCT	VGPR	PR	G-CSF	11.5
7	3 cycles VCD	PR	ASCT	VGPR	VGPR	G-CSF + Plerixafor	8.35
8	3 cycles VCD	CR	ASCT	CR	VGPR	G-CSF	8.56
9	4 cycles VCD	CR	ASCT	CR	CR	G-CSF	7.94
10	4 cycles VCD	CR	ASCT	CR	CR	G-CSF	8.46
11	4 cycles VCD	CR	ASCT	CR	CR	G-CSF	7.25
12	7 cycles VDT ^2^	VGPR	AlloSCT	CR	CR		
13	4 cycles VMP ^3^	VGPR	-	-	PR		
14	4 cycles VCD	PR	Lenalidomide	VGPR	VGPR		
15	3 cycles VMP	PR	Bortezomib	VGPR	VGPR		

**Table 5:** The relationship between mobilization and CD27, CD81, IL-10, and VEGF levels at diagnosis (Spearman’s rho).

Mobilization	CD27	CD81	IL-10	VEGF
Correlation coefficient	−0.753	−0.360	0.123	0.645
Sig. (2-tailed)	0.007	0.277	0.719	0.032

IL-10: Interleukin-10; VEGF: vascular endothelial growth factor.

**Table 6:** Comparison of IL-10 and VEGF values obtained from patients at diagnosis with healthy control group.

	Median IL-10 (pg/mL)	*p* Value *	Median VEGF (pg/mL)	*p* Value *
Patients at diagnosis (*n* = 35)	9.24	0.061	251.35	0.575
Healthy control group (*n* = 15)	4.91		252.12	

IL-10: Interleukin-10; VEGF: vascular endothelial growth factor; * *p* value was obtained using the Mann–Whitney U test.

**Table 7:** Comparison of IL-10 and VEGF values at diagnosis and in patients followed up after treatment.

Completely Followed Patients	Median IL-10 (pg/mL)	*p* Value *	Median VEGF (pg/mL)	*p* Value *
At diagnosis (*n* = 15)	7.72	0.681	282.70	0.001
After treatment (*n* = 15)	6.22		254.98	

IL-10: Interleukin-10; VEGF: vascular endothelial growth factor; * *p* value was obtained using the Wilcoxon test.

## Data Availability

The datasets utilized and/or examined in the present investigation are accessible from the corresponding author upon an appropriate request.
